# IFITM proteins drive type 2 T helper cell differentiation and exacerbate allergic airway inflammation

**DOI:** 10.1002/eji.201847692

**Published:** 2018-11-09

**Authors:** Diana C. Yánez, Hemant Sahni, Susan Ross, Anisha Solanki, Ching‐In Lau, Eleftheria Papaioannou, Alessandro Barbarulo, Rebecca Powell, Ulrike C. Lange, David J. Adams, Martino Barenco, Masahiro Ono, Fulvio D'Acquisto, Anna L. Furmanski, Tessa Crompton

**Affiliations:** ^1^ UCL Great Ormond Street Institute of Child Health London UK; ^2^ Department of Anesthesiology University Medical Center Hamburg‐Eppendorf Hamburg Germany; ^3^ The Wellcome Trust/Cancer Research UK Gurdon Institute Cambridge UK; ^4^ Wellcome Trust Sanger Institute Wellcome Trust Genome Campus Cambridge UK; ^5^ Department of Life Sciences, Sir Alexander Fleming Building Imperial College London London UK; ^6^ Health Science Research Centre University of Roehampton London UK; ^7^ School of Life Sciences University of Bedfordshire Luton UK; ^8^ School of Medicine Universidad San Francisco de Quito Quito Ecuador

**Keywords:** Allergic airway disease, IFN‐γ, Interferon‐inducible transmembrane (IFITM) protein, T helper 1 (Th1), T helper 2 (Th2)

## Abstract

The interferon‐inducible transmembrane (*Ifitm*/*Fragilis*) genes encode homologous proteins that are induced by IFNs. Here, we show that IFITM proteins regulate murine CD4^+^ Th cell differentiation. *Ifitm2* and *Ifitm3* are expressed in wild‐type (WT) CD4^+^ T cells. On activation, *Ifitm3* was downregulated and *Ifitm2* was upregulated. Resting *Ifitm*‐family‐deficient CD4^+^ T cells had higher expression of Th1‐associated genes than WT and purified naive *Ifitm*‐family‐deficient CD4^+^ T cells differentiated more efficiently to Th1, whereas Th2 differentiation was inhibited.

*Ifitm*‐family‐deficient mice, but not *Ifitm3*‐deficient mice, were less susceptible than WT to induction of allergic airways disease, with a weaker Th2 response and less severe disease and lower *Il4* but higher *Ifng* expression and IL‐27 secretion. Thus, the *Ifitm* family is important in adaptive immunity, influencing Th1/Th2 polarization, and Th2 immunopathology.

## Introduction

The family of interferon‐inducible transmembrane (*Ifitm*/*Fragilis*) genes (*Ifitm1*, *2*, *3*, *5*, and *6*) encode homologous proteins that have recently been shown to confer cellular resistance to viruses 1. Their promoters have one or more interferon stimulated response elements (ISRE), which make them responsive to IFNs 2. The IFITM family are also associated with germ cell specification during mouse embryonic development 3, 4, 5, and may play a role in the immune system, as in human cells, early cross‐linking antibody studies suggested that the IFITM1 protein (Leu13) was involved in T‐cell activation 6.

In tissue culture experiments, IFITM proteins enable cells to resist infection by both enveloped and nonenveloped viruses, and several distinct mechanisms have been proposed to explain their ability to increase cellular resistance to different viruses 1, 7, 8, 9, 10, 11.

In vivo studies showed that *Ifitm3* ^–/–^ confers resistance to influenza infection in both humans and mice 12, 13, 14, 15 but the role of IFITM proteins in adaptive immunity per se has not been explored. Here, we investigate the function of the IFITM family in CD4^+^ Th cell differentiation. We show that *Ifitm2* is rapidly upregulated following T‐cell activation, and that IFITM proteins promote Th2 differentiation and inhibit Th1 differentiation in a T‐cell intrinsic manner. In the absence of IFITM proteins, the Th2 response is diminished in allergic airway disease, reducing the severity of disease, but deletion of *Ifitm3* alone does not reduce disease severity. These findings demonstrate an important role for IFITM proteins in adaptive immunity, in the cell‐intrinsic regulation of CD4^+^ Th differentiation and fate.

## Results

### Absence of IFITM proteins favors a Th1 transcriptional profile

**Figure 1:**
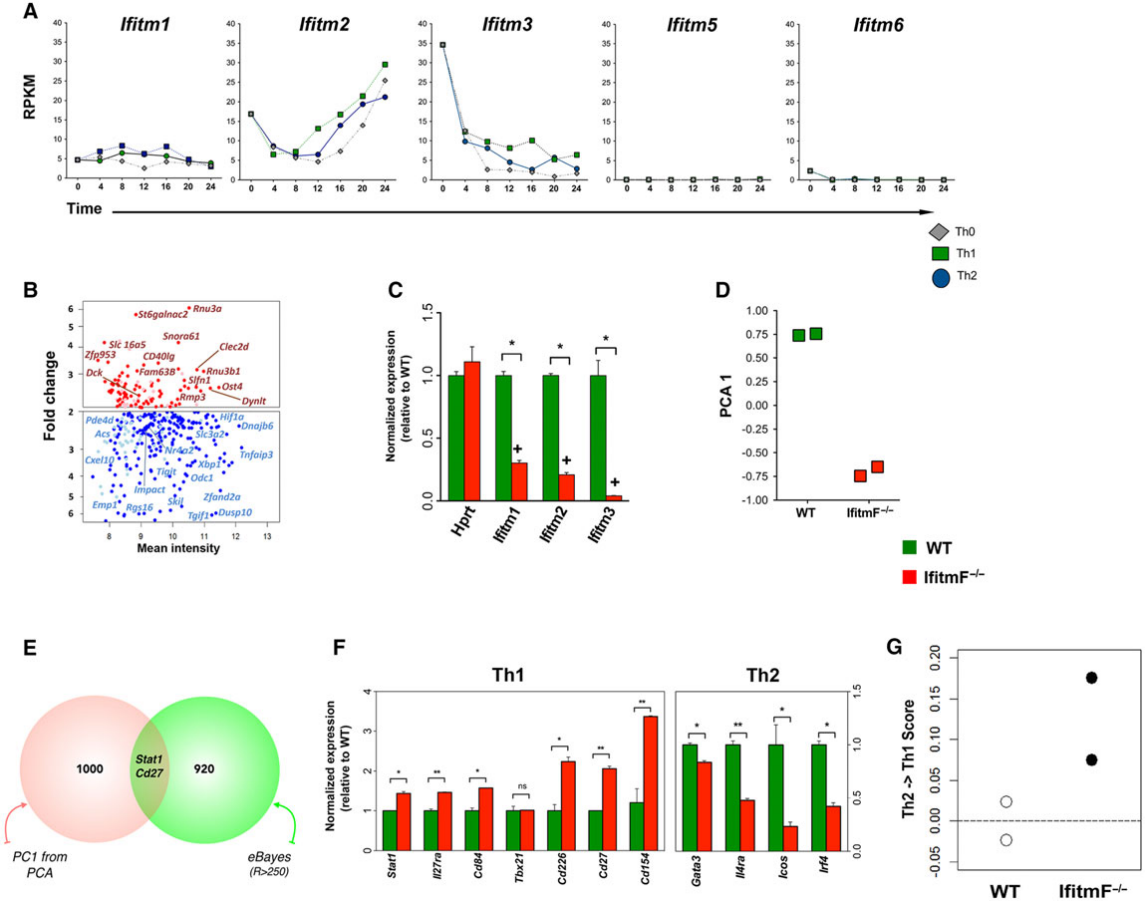
Absence of IFITM proteins biases resting CD4^+^ T cells to a Th1‐like transcriptional profile. (A) RNAseq was carried out on purified CD4^+^ T cells from WT spleen pooled from six mice, activated with anti‐CD3 and anti‐CD28 in skewing conditions, and cells were removed from the cultures for RNA sequencing at 4 h time points after activation. Each different time point and culture conditions combination was sequenced once to generate one dataset. Graphs show expression (RPKM). (B–F) Affymetrix microarray analysis was carried out on purified CD4^+^ T cells from WT and *IfitmF* ^–/–^ spleen. Two datasets were obtained for each genotype from biological replicates and separate purifications. Cells purified from two mice were pooled for each dataset. (B) Scatter plot: The relative change in expression (fold change) of significantly different genes (DEG with *p* < 0.05 by eBayes, FDR adjusted, with a fold change greater than two) in *IfitmF* ^–/–^ compared to WT, plotted against the mean intensity (RMA normalized value, plotted on a log2 scale). Selected genes have been marked on the plot. Red/blue dots represent increased/decreased expression in the *IfitmF* ^–/–^ compared to WT; light red/light blue dots represent probes not annotated to any gene on the Affymetrix 1.0 ST platform. Both the axes are on a log2 scale with the labels in the *y*‐axis representing the nonlogarithmic relative fold changes in KO relative to WT mice. (C) Relative expression (nonlog RMA normalized value, relative to mean of WT) in *IfitmF* ^–/–^ and WT CD4^+^ T cells. (Histograms show mean ± SEM; + represents expression below detection threshold. *p < 0.05 by eBayes, FDR adjusted value. (D) Datasets for WT and *IfitmF* ^–/–^ plotted on PC1. (E) The 1000 genes that contributed most to PC1 were intersected with 920 DEG with a range of at least 250 units (nonlog RMA normalized) to identify genes‐of‐interest, including *Stat1* and *Cd27*. These genes are listed in supporting Information Table 1. (F) Normalized expression (nonlog RMA normalized value, relative to mean of WT) in the *IfitmF* ^–/–^ and WT CD4^+^ T cells of the Th1 (left) and Th2 (right) associated genes listed. (Bar charts show mean ± SEM; *p < 0.05; **p < 0.01, Student's *t*‐test). (G) CCA was used to plot samples on a scale of Th2→Th1 scale. The scale was generated from Affymetrix datasets from in vitro‐skewed Th2 and Th1 cells. On the scale, 0 is the mean value of WT samples; Th1 are positive.

To explore a possible function for the IFITM family in CD4^+^ T‐cell activation or differentiation, we first measured expression of the *Ifitm* genes by RNA sequencing from CD4^+^ T cells for a 24 h time course following in vitro activation with anti‐CD3 and anti‐CD28 in Th0/Th1/Th2 skewing‐conditions (GEO: GSE93915; Fig. 1A). This time‐course analysis showed that *Ifitm1* was expressed at low levels throughout the time course in all conditions. At the start of the experiment, *Ifitm3* was most highly expressed of the three genes, but it was then rapidly downregulated after 4 h in response to the TCR/CD28 stimulus. In contrast, after an initial downregulation, expression of *Ifitm2* increased to above resting levels, with highest expression overall in Th1 skewing conditions. Expression of all three *Ifitm1–3* genes was lower in the Th2 culture conditions than Th0 and Th1 conditions from 4 h after stimulation onwards, consistent with the fact that they are IFN response genes, and that the Th2 skewing culture conditions include an anti‐IFN‐γ mab. *Ifitm5* was below detection, whereas *Ifitm6* was expressed at very low levels in resting CD4^+^ T cells and rapidly downregulated after 4 h to below detection levels in all culture conditions.

Given the expression patterns of *Ifitm2* and *Ifitm3* in CD4^+^ T‐cells in response to TCR/CD28 ligation, we tested if the IFITM family are involved in CD4^+^ T‐cell activation in vitro, but on anti‐CD3/CD28 stimulation, we found no differences in expression of activation markers or in proliferation between WT CD4^+^ T‐cells and IFITM‐deficient CD4^+^ T cells (from mice in which the entire *Ifitm* gene family had been deleted [*IfitmF* ^–/–^]) (Supporting Information Fig. 1A–D).

Therefore, to investigate further the function of these genes in CD4^+^ T cells, we carried out whole genome transcriptome analysis on purified CD4^+^ T cells from spleen of *IfitmF* ^–/–^ and WT to identify common effects of *Ifitm* genes in resting CD4^+^ T cells (GEO: GSE90494). We identified 920 differentially expressed genes (DEG) between WT and *IfitmF* ^–/–^ (Fig. 1B). As expected, these included the *Ifitm* family members, *Ifitm1*, *Ifitm2*, and *Ifitm3*, which were below detection threshold in the knockout (Fig. 1C).

In order to explore the molecular variability between the two genotypes in an unbiased way, we carried out principal component analysis. The first principal component axis (PC1), which accounted for 52.7% of variability within the dataset, separated the samples by genotype (Fig. 1D). We then identified genes‐of‐interest, by intersecting the 1000 genes that contributed most to PC1 with the 920 DEG (Fig. 1E; Supporting Information Table 1). This intersection included genes for the costimulatory molecule CD27, which can sensitize naïve T cells to Th1 differentiation through interactions with CD70 on APC, and the transcription factor Stat1, which plays an important role in IFN‐γ signal transduction 16, 17 (Fig. 1E). Given that *Cd27* and *Stat1* are both involved in the Th1 response, we examined expression of other genes known to be associated with Th1 or Th2 responses 18. We found significant increase in expression in the *IfitmF* ^–/–^ CD4^+^ T cells compared to WT of Th1‐associated genes such as *Cd84*, *Cd226*, and *Cd154* and also *Il27ra*, which signals for Stat1 activation during early Th1 differentiation 19, 20, 21, whereas there was no difference in expression levels of the master Th1 transcription factor *Tbx21* between genotypes (Fig. 1F). Interestingly, expression of the Th2‐associated genes *Il4ra*, *ICOS*, *Irf4*, and the Th2‐transcription factor *Gata3* were significantly lower in the *IfitmF* ^–/–^ cells (Fig. 1F). Thus, the transcriptome data suggested an overall reduction in expression of Th2‐associated genes and a tendency toward increased expression of Th1‐associated genes in the *IfitmF* ^–/–^ CD4^+^ T cells compared to WT (Fig. 1F).

To test this idea on a wider gene set, we generated a scale of Th2‐to‐Th1 skewedness (Th2→Th1 score) and plotted our datasets against this scale using canonical correspondence analysis (CCA), as previously described 22. The scale was generated from the thousand most DEG (by eBayes) between Th1 and Th2 CD4^+^ T cells in publically available whole genome transcriptome data derived from Th‐skewed cultures (GEO: GSE14308). Interestingly, although the reference data were obtained from T cells that had been cultured for several days in skewing conditions, *IfitmF* ^–/–^ CD4^+^ T cells showed a clear bias toward the Th1 transcriptional profile compared to the WT (Fig. 1G). Thus, these analyses suggested that absence of the IFITM family of proteins predisposed resting CD4^+^ T cells to Th1 differentiation in the spleen.

### IFITM proteins polarize Th differentiation in vitro

**Figure 2:**
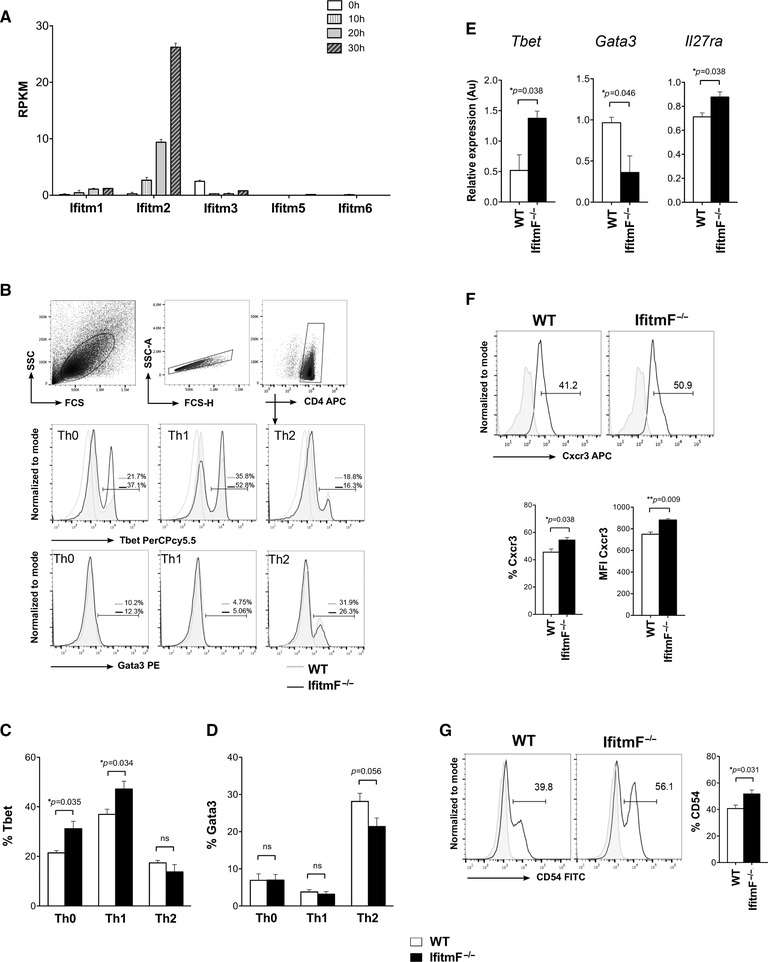
Absence of IFITM proteins biases CD4^+^ T cells to Th1 in vitro. (A) Expression (RPKM) by RNAseq of *Ifitm* genes in naïve CD4^+^ T cells from WT splenocytes, activated with anti‐CD3/CD28. Two independent datasets were obtained for each time point from separate FACS sorts (*n* = 7 WT mice). Data are shown as mean ± SEM. (B) Flow cytometry analysis of intracellular staining of Tbet and Gata3 from WT and *IfitmF* ^–/–^ on purified naïve CD4^+^ T cells cultured for 72 h in Th0, Th1 and Th2 conditions. Dot plots show the gating strategy to identify live CD4^+^ T cells at the end of the culture period. Histograms show the percentage of positive cells in the marker for *IfitmF* ^–/–^ (black line) and WT (grey line). Naïve CD4^+^ T cells were purified and cells from each individual mouse were cultured independently (WT *n* = 4 and *IfitmF* ^–/–^ *n* = 4 mice per experiment). (C) Percentage of Tbet^+^ CD4^+^ T cells cultured in the three different polarizing conditions from WT and *IfitmF* ^–/–^ mice (*n* = 4 mice per group). Data are shown as mean ± SEM and are representative of two independent experiments. (D) Percentage of Gata3^+^ cells in CD4^+^ T cells cultured in the three different conditions from WT and *IfitmF* ^–/–^ mice (*n* = 6 mice per group). Data are shown as mean ± SEM and are representative of two independent experiments. (E) qRT‐PCR after 72 h of culture of purified naïve CD4^+^ T cells for expression of *Tbet* and *Il27ra* in Th1 conditions and *Gata3* in Th2 conditions. Units are arbitrary (Au). Data are shown as mean ± SEM from three independent experiments (*n* = 3 mice for each genotype per experiment). (F) Histograms show cell surface expression of Cxcr3 under Th1 culture conditions for WT and *IfitmF* ^–/–^ CD4^+^ T cells. Bar charts show mean ± SEM percentage of cells in the marker shown, and the MFI of cells in the marker shown from CD4^+^ T cells from WT (*n* = 3 mice) and *IfitmF* ^–/–^ (*n* = 3 mice). Data shown are representative of three independent experiments. (G) (Left) Cell surface expression of CD54 from Th1 cultures of CD4^+^ T cells from WT and *IfitmF* ^–/–^. Data shown are representative of four independent experiments, (right) percentage of CD54^+^ cells gated on CD4^+^ T cells from the cultures from WT (*n* = 4 mice) and *IfitmF* ^–/–^ (*n* = 4 mice). Data shown are representative of two independent experiments. Grey histograms show staining with an isotype control antibody *p < 0.05, Student's *t*‐test.

Given the function significance of IFITM family deletion on the transcriptome of resting CD4^+^ T cells, we next investigated *Ifitm* expression in FACS‐sorted naïve WT CD4^+^ T cells by RNA sequencing, after anti‐CD3/CD28 activation over a longer 30‐h time course (Fig. 2A). At 30 h after activation, expression of *Ifitm2* was more than tenfold higher than *Ifitm1* and *Ifitm3*. As expected, expressions *Ifitm5* and *Ifitm6* were very low.

To test whether the IFITM proteins influence Th differentiation in a T‐cell intrinsic manner, we FACS‐sorted naïve CD4^+^ T cells from *IfitmF* ^–/–^ and WT littermate mice and activated them with anti‐CD3 and anti‐CD28 in the different skewing conditions (Fig. 2B). After 72 h in culture, intracellular Tbet expression was increased in the *IfitmF* ^–/–^ Th0 cultures, compared to WT control, suggesting a cell‐intrinsic bias of naïve CD4^+^ T cells toward Th1 differentiation in the absence of IFITM proteins. In Th1 skewing conditions, the percentage of Tbet^+^ cells was also significantly higher in *IfitmF* ^–/–^ compared to WT CD4^+^ T cells, showing enhanced commitment to the Th1 lineage, whereas there was no difference in Tbet expression under Th2 condition (Fig. 2B–C). In contrast, expression of the Th2‐transcription factor Gata3 was low and equivalent between WT and *IfitmF* ^–/–^ CD4^+^ T cells cultured in Th0 and Th1 conditions, and was lower in *IfitmF* ^–/–^ compared to WT CD4^+^ T cells cultured in Th2 conditions (Fig. 2B and D). Analysis of gene expression by quantitative RT‐PCR (qRT‐PCR) also showed that *Tbx21* (*Tbet*) was more highly expressed in *IfitmF* ^–/–^ Th1 cultures than in WT, whereas expression of *Gata3* was lower in *IfitmF* ^–/–^ Th2 cultures (Fig. 2E). We also observed a modest but significant increase in expression of *Il27ra* in the Th1‐skewed *IfitmF* ^–/–^ cultures compared to WT (Fig. 2E), consistent with the microarray analysis of resting CD4^+^ T‐cells (Fig. 1F). Supporting the Th1 profile, cell surface expression of the Th1 marker, Cxcr3, was increased in Th1‐skewing cultures cells from *IfitmF* ^–/–^ mice compared to WT after 72 h of culture, and likewise there was an increase in cell surface expression of CD54 (Icam1), which on naive CD4^+^ T cells plays an important role in the early stages of Th1 differentiation by regulation of IL‐27 (Fig. 2F–G) 23.

**Figure 3:**
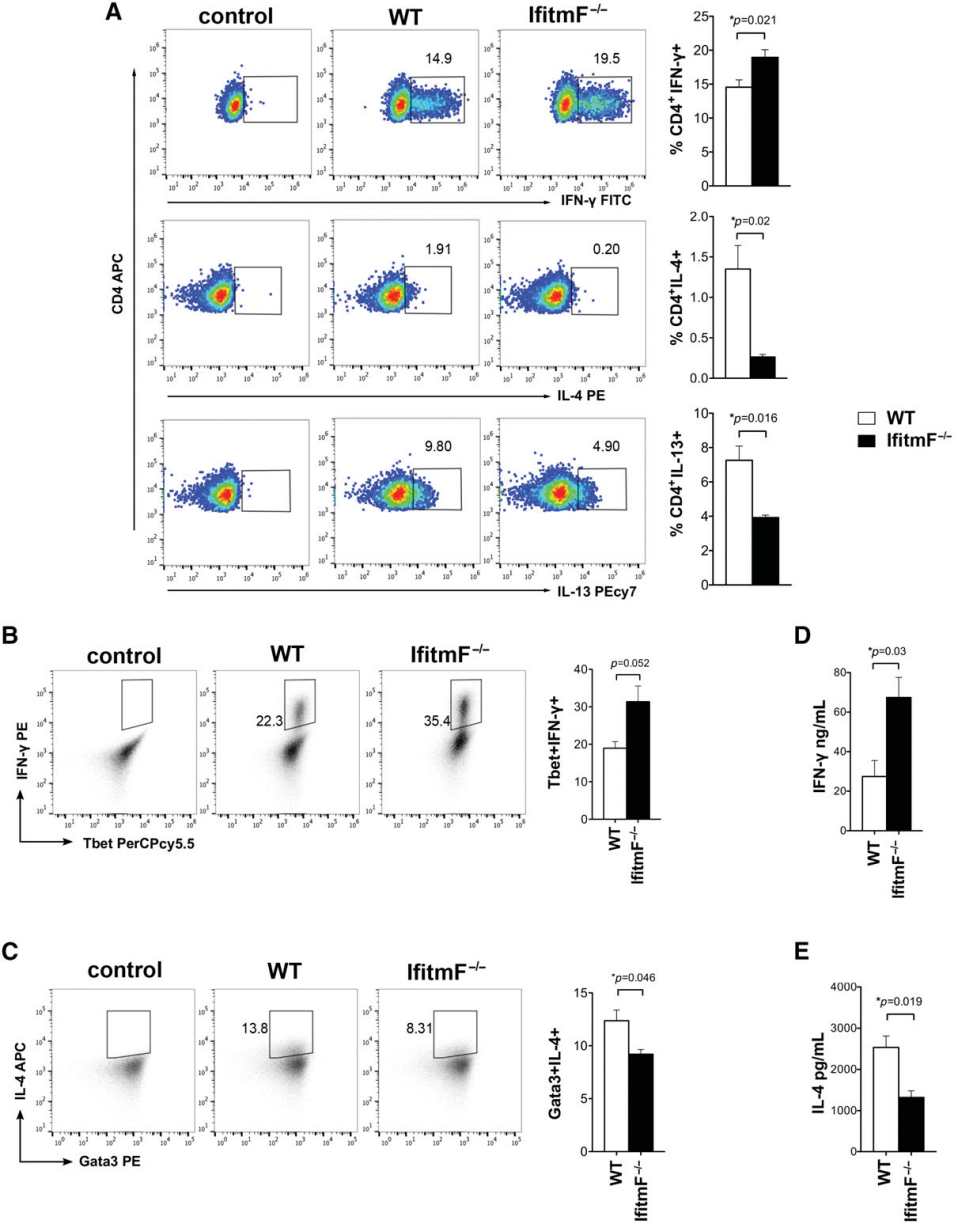
The absence of IFITM proteins promotes Th1 cytokine production and decreases Th2 cytokine production. (A) Purified naïve CD4^+^ T cells from *IfitmF* ^–/–^ and WT mice were cultured in vitro under Th1 and Th2 skewing conditions. Dot plots show the intracellular expression of IFN‐γ under Th1 conditions (above), IL‐4 (middle), and IL‐13 (bottom) under Th2 conditions in CD4^+^ T cells from WT and *IfitmF* ^–/–^ after 72 h of culture. Data shown are representative of three independent experiments. In each case, the control plots show negative control. Bar charts show mean ± SEM percentage of cells that stained positive for each cytokine, for IFN‐γ in *IfitmF* ^–/–^ (*n* = 6 mice) cultures compared to WT (*n* = 5 mice); and for IL‐13 and IL‐4 (*n* = 3 mice per genotype). (B, C) Dot plots, representative of three experiments, show the intracellular expression in CD4^+^ T cells from WT and *IfitmF* ^–/–^ cultured for 6 days and stimulated as described in (A), showing (B) Tbet^+^IFN‐γ^+^ in Th1 conditions and (C) Gata3^+^IL‐4^+^ in Th2 conditions. The control plots show negative control. Bar charts show mean ± SEM. (D) Concentration of IFN‐γ in supernatants from in vitro Th1 cultures after 6 days was measured by ELISA (*n* = 3 mice for each genotype). (E) Concentration of IL‐4 in supernatants from in vitro Th2 cultures after 6 days measured by ELISA. Data are shown as mean ± SEM (*n* = 3 samples) *p < 0.05, Student's *t*‐test.

The pattern of cytokine expression confirmed the Th1 bias of the *IfitmF* ^–/–^ deficient CD4^+^ T cells. After 3 days of culture in Th1 conditions, the proportion of CD4^+^ T cells that expressed the key Th1 cytokine IFN‐γ was significantly increased in *IfitmF* ^–/–^ CD4^+^ T cells compared to littermate WT (Fig. 3A), whereas in Th0 conditions, very few cells expressed intracellular IFN‐γ and there was no difference between genotypes (Supporting Information Fig. 1E). In contrast, the percentage of cells that expressed IL‐4 and IL‐13 was reduced in *IfitmF* ^–/–^ CD4^+^ T cells cultured for 3 days in Th2 conditions compared to WT (Fig. 3A).

After 6 days of culture in Th1 conditions, the percentage of Tbet^+^ IFN‐γ^+^ cells was also higher in *IfitmF* ^–/–^ CD4^+^ T cells compared to WT (Fig. 3B), whereas the percentage of Gata3^+^ IL‐4^+^ cells after 6 days of culture in Th2 conditions was lower in *IfitmF* ^–/–^ CD4^+^ T cells compared to WT (Fig. 3C). Measurement of cytokine secretion confirmed these findings, as the concentration of IFN‐γ was increased in supernatants from *IfitmF* ^–/–^ compared to WT cultures after 6 days of culture in Th1 conditions (Fig. 3D), but the concentration of IL‐4 was lower after 6 days of culture in Th2 conditions (Fig. 3E).

Thus, naïve CD4^+^ T cells purified from *IfitmF* ^–/–^ mice showed a cell‐intrinsic bias toward Th1 differentiation, with concomitant repression of Th2 differentiation. These data suggest a negative feedback loop between IFITM proteins and Th1‐differentiation in CD4^+^ T‐cells: *Ifitm* genes are induced by IFN‐γ, but in the absence of IFITMs, IFN‐γ expression and Th1 differentiation are favored.

### Absence of IFITM proteins reduces the Th2 response in a murine asthma model

Th1 cells can inhibit Th2‐induced inflammation in the lung, through production of IFN‐γ 24, 25. Since we found that the absence of IFITM proteins promoted Th1 differentiation, with an increase in IFN‐γ levels, we used a murine model of allergic airways disease to test whether the IFITM family is required for Th2‐induced effector functions in vivo. We used the allergen papain, which is widely used as an asthma model to induce allergic airway inflammation by promoting a Th2 response 26, 27, 28, 29.

**Figure 4:**
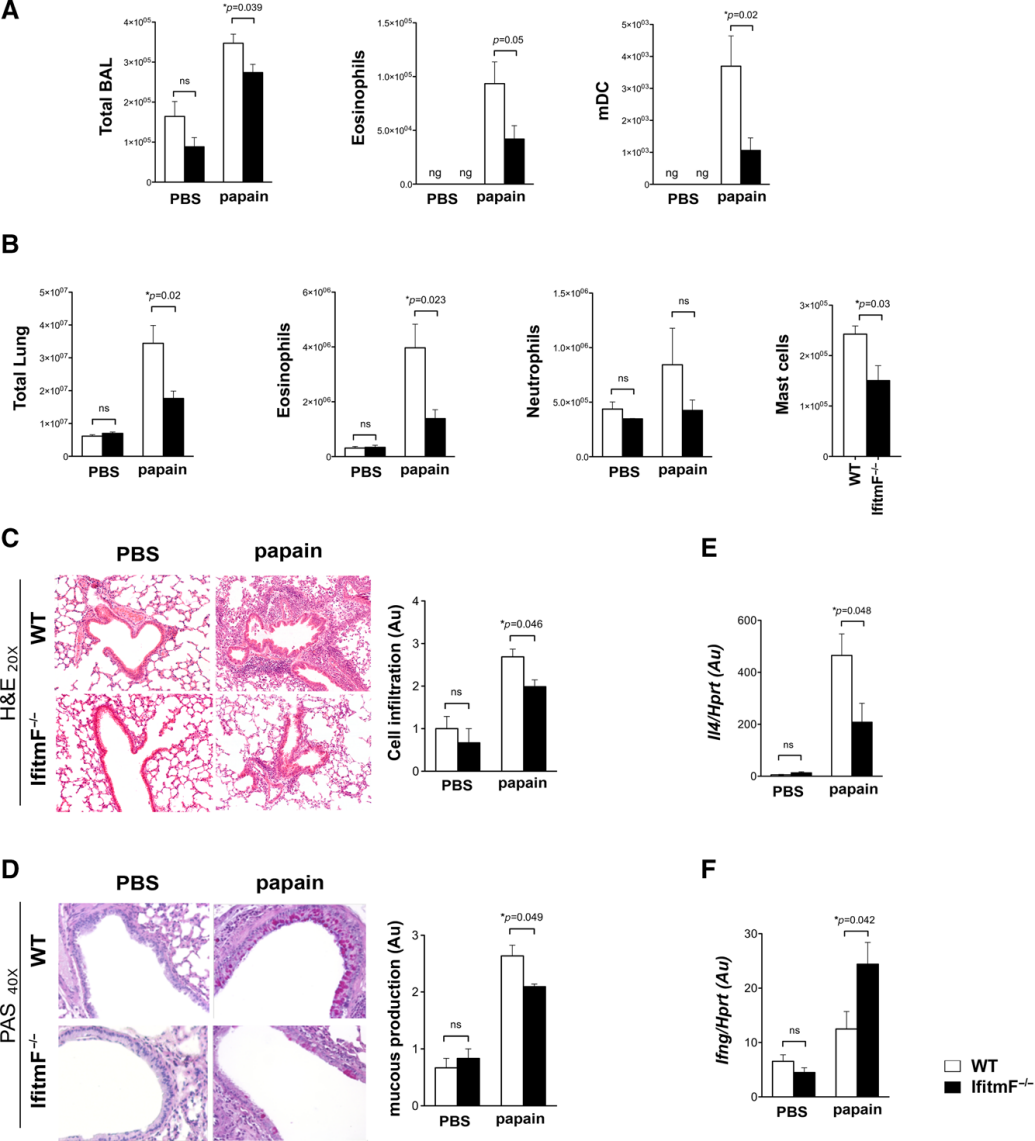
Absence of IFITM proteins decreases Th‐2 immunopathology. WT and *IfitmF* ^–/–^ mice received intranasal challenge with PBS or papain (described in the cartoon in Supporting Information Fig. 2A). (A) Bar charts show the mean ± SEM number of cells in BAL and mean number of eosinophils and mDC in BAL. (B) The mean number of cells recovered from lungs (total lung, left side) and mean number of eosinophils, mast cells, and neutrophils recovered from lung of WT and *IfitmF* ^–/–^ mice. (C, D) Representative examples of cellular infiltration and mucous production in formalin‐fixed paraffin‐embedded lung sections of WT (*n* = 3 mice) and *IfitmF* ^–/–^ (*n* = 3 mice) in PBS and papain conditions. A blind semiquantitative histological assessment was performed to score cellular infiltration by H&E staining and mucous production by PAS staining. (C) H&E staining: 20× magnification; bar chart shows scale of cellular infiltration of airways, from 0 to 4 (arbitrary units). (D) PAS staining: 40× magnification; bar chart shows scale of mucous production, from 0 to 3 (arbitrary units). (E, F) qRT‐PCR for expression of *Il4* (E) and (F) *Infg* in lung in PBS and papain conditions in WT (*n* = 6 mice) and *IfitmF* ^–/–^ (*n* = 6 mice). Au = arbitrary units. Bar charts represent mean ± SEM. Data in (A, B, E, and F) are representative of two independent experiments with five or six mice per genotype. ng, negligible; *p < 0.05, Student's *t*‐test.

After repeated intranasal administration of papain or phosphate‐buffered saline (PBS; Supporting Information Fig. 2A), bronchoalveolar lavage (BAL), lungs, and mediastinal lymph nodes (mLN) were collected from WT and *IfitmF* ^–/–^ mice. As expected, papain sensitization produced robust recruitment of inflammatory cells to the BAL and lungs in both WT and *IfitmF* ^–/–^ mice (Supporting Information Fig. 2B–C). The BAL from *IfitmF* ^–/–^ contained significantly fewer cells than BAL from WT mice and contained significantly fewer eosinophils and myeloid dendritic cells (mDC) than WT after papain challenge (Fig. 4A). The cellular infiltration and number of eosinophils and mast cells isolated from the lungs were also significantly lower in the *IfitmF* ^–/–^ mice than WT, reflecting decreased severity of the inflammatory phase of disease (Fig. 4B). We observed no significant difference in the numbers of neutrophils between the WT and *IfitmF* ^–/–^ lungs (Fig. 4B). Immunohistochemistry of lung tissue showed that the *IfitmF* ^–/–^ lungs also had significantly lower cellular infiltration than WT after papain treatment (Fig. 4C). Periodic acid–Schiff (PAS) staining showed that mucous production was suppressed in *IfitmF* ^–/–^ compared to WT lungs (Fig. 4D).

Consistent with the induction of allergic airways disease, *Il4* expression in lung was upregulated on papain sensitization (Fig. 4E). Expression of *Il4* mRNA was lower in the homogenized lungs of *IfitmF* ^–/–^ mice compared to WT, after papain challenge, consistent with weaker induction of disease (Fig. 4E). In contrast, *Ifng* expression was not significantly increased in WT lungs on papain sensitization, but was upregulated in *IfitmF* ^–/–^ lungs, and on papain treatment was significantly higher in *IfitmF* ^–/–^ than WT lung (Fig. 4F).

**Figure 5:**
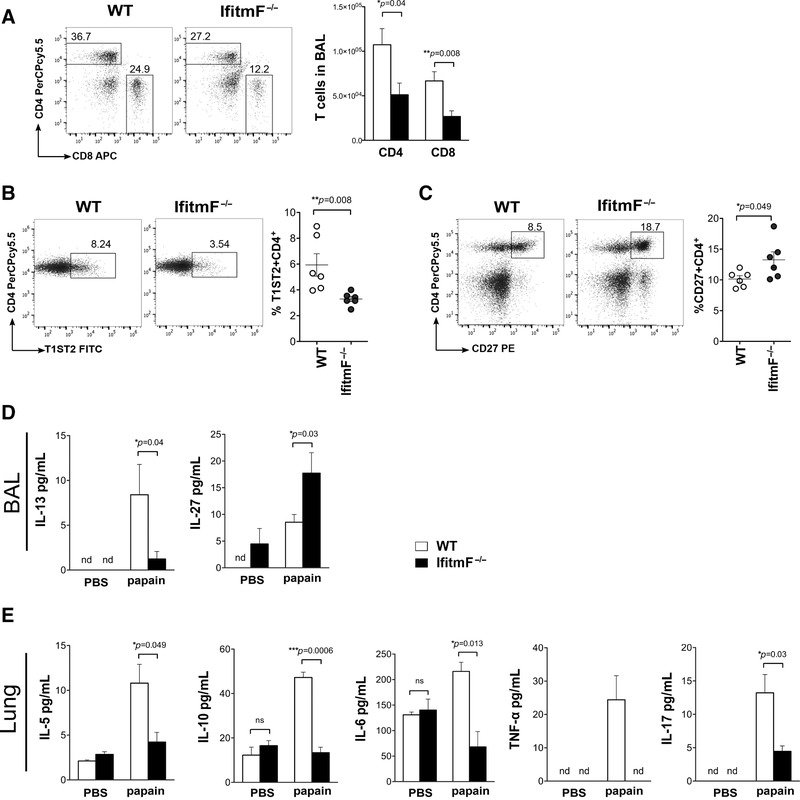
Effect of IFITM deficiency on the Th2 population and cytokine production in the papain asthma model. (A) Flow cytometry analysis of cells from BAL. Bar chart shows mean ±SEM number of CD4 and CD8 T cells in BAL from papain‐treated WT and *IfitmF* ^–/–^. (B) Dot plots show anti‐CD4 and T1ST2 staining, indicating the percentage of T1ST2^+^CD4^+^ T cells in the region shown from papain‐treated WT and *IfitmF* ^–/–^ mLN. Scatter plots show mean ±SEM percentage of T1ST2^+^CD4^+^ positive cells. Each dot represents an individual mouse. (C) Frequencies of cells in the mLN from papain‐treated WT and *IfitmF* ^–/–^. Scatter plots show mean ± SEM percentage of CD27^+^CD4^+^ cells in mLN from papain‐treated WT and *IfitmF* ^–/–^. Each dot represents an individual mouse (*n* = 6 per genotype). (D) Cytokine levels in BAL of PBS versus papain‐treated WT (*n* = 6 mice) and *IfitmF* ^–/–^ mice (*n* = 6 mice). (K) Quantification of inflammatory cytokines in lung homogenates from WT (*n* = 3) and *IfitmF* ^–/–^ (*n* = 3) mice in PBS and papain conditions, error bars ± SEM. (A–C) Data are representative of two independent experiments with five to six mice per group in each. nd, not detectable, *p < 0.05; **p < 0.01; ***p < 0.001, Student's *t*‐test.

In addition to the decrease in inflammatory cells in *IfitmF* ^–/–^ lungs and BAL compared to WT, we observed a significant decrease in the recruitment of CD4 and CD8 T cells in BAL in *IfitmF* ^–/–^ compared to WT (Fig. 5A). There was a decrease in the percentage of cells that expressed cell surface T1ST2, a marker of activated Th2 cells 30 in mLN from papain‐sensitized *IfitmF* ^–/–^ compared to WT (Fig. 5B). Cell surface CD27 expression was significantly increased on mLN CD4^+^ T cells from papain‐treated *IfitmF* ^–/–^ compared to WT (Fig. 5C), consistent with the higher expression of *Cd27* in resting CD4^+^ T cells from the *IfitmF* ^–/–^ spleen compared to WT (Fig. 1F).

We then examined cytokine production on papain treatment. The concentration of the Th2 cytokine IL‐13 was significantly lower in BAL from *IfitmF* ^–/–^ than WT (Fig. 5D). Interestingly, the concentration of IL‐27, which can promote IFN‐γ production through activation of Stat1, was higher in the *IfitmF* ^–/–^ BAL (Fig. 5D). In lung homogenates, the Th2 cytokines, IL‐5, IL‐10, and IL‐6 were lower in *IfitmF* ^–/–^ than WT (Fig. 5E). In addition, there were lower concentrations of proinflammatory cytokines TNF‐α and IL‐17 in *IfitmF* ^–/–^ lungs compared to WT after papain treatment (Fig. 5E). Thus, absence of the IFITM family reduced the Th2‐inflammatory effect in allergic airway disease.

### Ifitm3 deletion does not reduce the Th2 response

As both *Ifitm2* and *Ifitm3* are regulated by TCR/CD28 ligation and are more highly expressed in Th1‐skewing conditions than neutral or Th2‐skewing conditions (Figs. 1A and 2A), it is possible that it is the absence of either *Ifitm2* or *Ifitm3* that influences Th1/2 differentiation in the *IfitmF* ^–/–^ mice.

**Figure 6:**
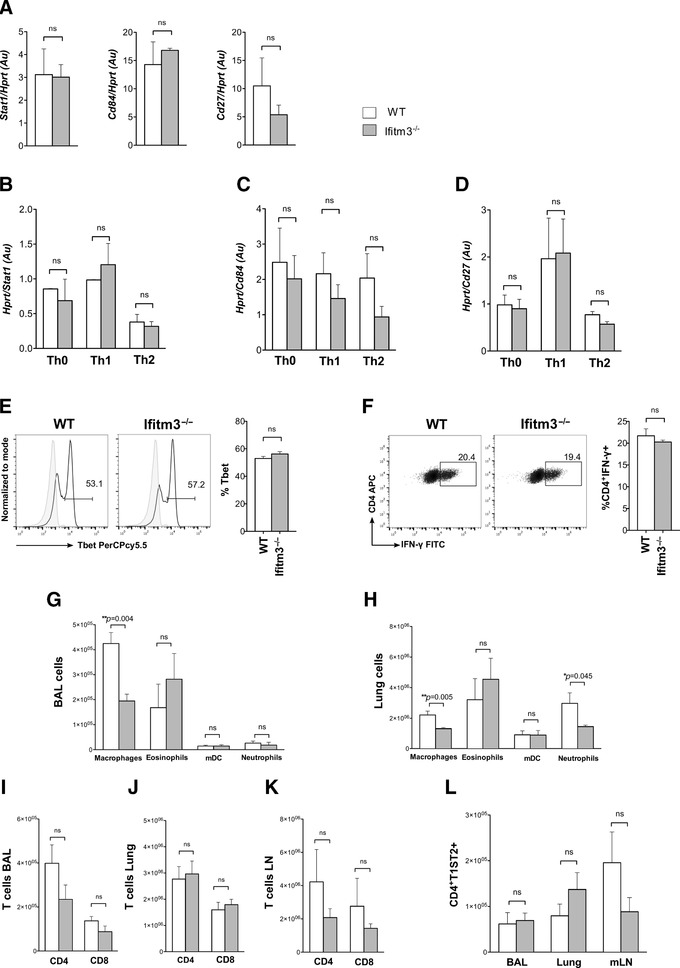
Effect of IFITM3 on CD4^+^ T cell differentiation. (A–D) Relative expression of *Stat1*, *Cd84*, and *Cd27* were measured by qRT‐PCR of purified CD4^+^ T cells in (A) resting conditions and (B–D) in CD4^+^ T cells cultured for 72 h in skewing conditions of WT and *Ifitm3* ^–/–^ mice. Bar charts show mean expression ± SEM, relative to *Hprt*, units are arbitrary (Au). The bar charts show mean from three separate experiments carried out with biological replicates. (E) Histogram shows intracellular Tbet protein expression in WT and *Ifitm3* ^–/–^ naïve CD4^+^ T cells in Th1 skewing conditions cultured for 72 h from a representative experiment of 3, for WT (*n* = 3 mice) and *Ifitm3* ^–/–^ (*n* = 3 mice). Bar charts show mean ± SEM percentage of cells that stained positive for Tbet in WT and *IfitmF* ^–/–^. (F) Dot plots show intracellular IFN‐γ expression in purified naïve CD4^+^ T cells from *Ifitm3* ^–/–^ and WT under Th1 conditions for 72 h from a representative experiment of WT (*n* = 3 mice) and *Ifitm3* ^–/–^ (*n* = 3 mice). Bar chart shows mean percentage of CD4^+^IFN‐γ^+^ cells. (G–L) WT (*n* = 4) and *Ifitm3* ^–/–^ (*n* = 4) mice underwent intranasal challenge with papain and PBS. (E and F) Bar charts show the mean ± SEM number of cells in BAL and lungs: macrophages (CD11b^low^CD11c^+^FSC^int^SSC^int^); eosinophils (CD11b^+^SiglecF^+^FSC^low^SSC^int^); mDC (Gr1^−^CD317^−^CD11b^+^CD11c^+^) and neutrophils (CD11b^hi^CD11c^−^Ly6g^+^ FSC^low^SSC^low^). Bar charts show cell number of CD4, and CD8 T cells in (I) BAL, (J) lungs, (K) mLN in WT, and *Ifitm3* ^–/–^ mice. (L) Quantification of T1ST2 in CD4^+^ T cells from BAL, lung, and mLN. Error bars ± SEM.

We therefore assessed expression of Th1‐associated genes that we had identified to be differentially expressed in CD4^+^ T cells from *IfitmF* ^–/–^ spleen, in CD4^+^ T cells isolated from *Ifitm3* ^–/–^ and WT spleen. We found no significant differences in expression of *Stat1*, *Cd84*, or *Cd27* between *Ifitm3* ^–/–^ and WT CD4^+^ T cells purified fresh from the spleen (Fig. 6A) or cultured in skewing conditions after 72 h (Fig. 6B–D). Likewise, there was no significant difference in the proportion of *Ifitm3* ^–/–^ CD4^+^ T cells that stained positive with intracellular anti‐Tbet and anti‐IFN‐γ after 72‐h activation of purified naive CD4^+^ T cells in Th1 skewing conditions, compared to WT counterparts (Fig. 6E and F). This experiment together with the gene expression studies suggests that deletion of *Ifitm3* alone does not predispose CD4^+^ T cells to Th1 differentiation. To confirm this in vivo, we induced allergic airways disease by intranasal papain administration, and monitored cell infiltration into the lungs and BAL. There were no significant differences in the numbers of eosinophils, DC, and neutrophils in the BAL and lung. In contrast to the papain‐treated *IfitmF* ^–/–^ mice, we did, however, find a significant reduction in the number of macrophages in the *Ifitm3* ^–/–^ BAL and of macrophages and neutrophils in the papain‐treated *Ifitm3* ^–/–^ lung compared to WT (Fig. 6G–H). Finally, there were not significant differences in the number of T cells (Fig. 6I–K) or in the number of CD4^+^T1ST2^+^ cells (Fig. 6L) in BAL, lung, or mLN. Overall, we found no evidence for Th1 bias in *Ifitm3* ^–/–^ mice, whereas deletion of the *Ifitm* family reduced induction of allergic airways disease and the Th2 response.

## Discussion

Here, we show that deletion of the *Ifitm* family biases CD4^+^ Th differentiation to Th1 and reduces the severity of allergic airways disease in a murine model of allergic asthma. Much recent research on IFITM proteins has focused on their role in cellular resistance to viral infections, but their function in CD4^+^ T cells during the adaptive immune response is less well understood.

We showed that *Ifitm1*, *2*, and *3* are expressed in peripheral T cells, and that expression of *Ifitm2* and *Ifitm3* are differentially regulated by TCR/CD28 ligation, with rapid downregulation of *Ifitm3* but upregulation of *Ifitm2*. In mice deficient in the *Ifitm* family, resting CD4^+^ T cells had a transcriptional signature closer to that of Th1 cells than WT, and sorted naïve CD4^+^ T cells were polarized toward Th1 differentiation in a cell‐autonomous manner when cultured in vitro in Th‐neutral and Th1 skewing conditions. IFN‐γ production was increased in vitro and on induction of allergic airways disease, whereas Th2 responses were reduced. Thus, the IFITM family seems to provide a negative feedback loop on Th1 differentiation in CD4^+^ T cells, as they are IFN‐inducible proteins, which function to limit Th1 differentiation.

Taken together, our experiments suggest that one or more of the IFITM family of proteins act as negative regulators of Th1 differentiation and influence Th1/Th2 polarization. As the RNA‐sequencing data showed that *Ifitm2* expression increased in CD4^+^ T cells during the first 30 h following activation, whereas *Ifitm3* expression was rapidly downregulated, IFITM2 is the most likely candidate. In support of this, we found no impact of *Ifitm3* deficiency alone on IFN‐γ production or Th1 differentiation, and on induction of allergic airways disease; there was no significant impact of *Ifitm3* deficiency on Th2 effector populations or on eosinophil or mast cell infiltration of the lung. Therefore, these experiments also indicate that *Ifitm2* is most likely the key family member that is required for Th2 differentiation and inhibition of Th1 differentiation, although we cannot exclude a synergistic or additive effect between IFITM proteins.

Genome‐wide transcriptome analysis on purified CD4^+^ T cells from the spleen of the *IfitmF* ^–/–^ and WT revealed insights into causes of the enhanced Th1 response. Amongst DEG, we found the Th1 transcription factor *Stat1*, which was significantly higher in the *IfitmF* ^–/–^, as was transcription of other Th1‐associated genes, such as *Il27ra* (WSX‐1, Gp130), *Cd27*, and *Cd84*. Interestingly, we detected no difference in *Tbx21* (*Tbet*) transcription in resting CD4^+^ T cells, but Tbet expression was increased on activation in *IfitmF* ^–/–^ CD4^+^ T cells compared to WT. As IFITM proteins are transmembrane proteins that are found in intracellular and plasma membranes, the way in which their deletion has such a profound influence on the pattern of transcription is not immediately clear. However, membrane fusion studies have suggested that IFITM proteins block early steps of viral replication by influencing the molecular order of membranes and membrane fluidity 11. Interestingly, plasma membrane lipid order may also be an important factor that influences Th differentiation. High membrane order is related to Th2 differentiation, with higher production of IL‐4, whereas intermediate membrane order is present in Th1 cells secreting IFN‐γ^31^.

The absence of IFITM proteins may have a protective role in allergic diseases, such as asthma, by inhibition of the Th2 response, decreasing the severity of the inflammatory process. Several reports support the protective role of IFN‐γ in airway inflammation. IFN‐γ can inhibit Th2 cytokine effects in the airway and reduces production of eosinophils and mucus overproduction 24, 25. We found that the absence of IFITM family increased the level of *Ifng* in the lung, reduced cellular infiltration and Th2 cytokines, and attenuated inflammatory cytokine production.

In addition to its effect on Th1 differentiation, IL‐27 can play an anti‐inflammatory role in CD4^+^ T cells and is important in diseases induced by Th2 or Th17 cells 32, 33. IL‐27 can suppress the production of many different cytokines including IL‐4, IL‐5, IL‐13, IL‐2, IL‐6, IL‐10, TNF‐α, and IL‐17 34. Interestingly, although IL‐27 production was increased in the *IfitmF* ^–/–^ mice compared to WT in our asthma experiments, levels of *Il4*, IL‐5, IL‐13, IL‐6, IL‐10, IL17, and TNF‐α were all decreased.

Genome‐wide association studies (GWAS) have indicated that the Hedgehog (Hh) signaling pathway plays a role in allergic asthma 35, 36, 37, 38 and experimental mouse asthma studies have shown that increased Hh signaling to T cells and eosinophils exacerbates the disease 39, 40. Consistent with the reduction in allergic airways disease observed in the *IfitmF*‐deficient mice, both *Ifitm2* and *Ifitm3* are Hh‐target genes in CD4^+^ T cells 22. In the future, it will be interesting to dissect the interactions between Hh pathway activation and IFITM function in asthma.

Many studies have shown that the IFITM family provides cellular resistance to viral infection in vitro, and GWAS identified *IFITM3* as a resistance gene for influenza in humans 13, 41. However, we show here that deletion of IFITM proteins reduces the severity of Th2 inflammation in a mouse asthma model, suggesting an evolutionary balance between conferring cellular viral resistance and susceptibility to allergic disease. This study demonstrates that the IFITM family of proteins should be viewed not only as essential proteins for cellular resistance to viral infection but also as important regulators of CD4^+^ Th cell differentiation and function.

## Materials and methods

### Mice

*Ifitm*‐*family*‐deficient (*IfitmF* ^–/–^) and *Ifitm3*‐deficient (*Ifitm3* ^–/–^) mice were as described on a C57BL/6 background 2. Mice were bred and maintained at UCL. All mice studies were reviewed and approved by the British Home Office.

### Flow cytometry

Cells were stained as described 42 using antibodies from eBioscience or MD Biosciences and analyzed on a C6 Accuri flow cytometer (BD, Switzerland). Live cells were gated according to FSC/SSC profiles (as illustrated in Fig. 2B). For intracellular cytokine staining, cells were stimulated with PMA (50 ng/mL; Sigma, Sigma‐Aldrich, USA), Ionomycin (1 μg/mL; Sigma), and BrefeldinA (eBioscience) incubated in RPMI at 37°C for 4 h. Then, cells were fixed (Cytofix) and permeabilized (Bioscience) to carry out the intracellular staining for IFN‐γ, IL‐4, and IL‐13. Anti‐Tbet and anti‐Gata3 were staining as described 39 after fixation and permeabilization (antibodies/protein: eBioscience San Diego, CA, USA).

### Cell cultures

For activation, splenocytes at 5 × 10^6^ cells/mL were cultured in AIM‐V medium (Invitrogen, USA) with 10–5 M β‐mercaptoethanol and anti‐CD3/anti‐CD28 (BD Pharmingen, USA) at 0.01 μg/mL each. Cells were harvested at 4 and 20 h for analysis CD69 and CD25. Carboxyfluorescein diacetate succinimidyl ester (CFSE) labeling was carried out on splenocytes as previously described 22, then cells stimulated with anti‐CD3 and anti‐CD28 antibodies at 0.01 μg/mL and analyzed after 72 h.

For in vitro Th skewing experiments, splenocytes were treated with RBC lysis buffer before CD4^+^ T cells and pooled lymph nodes were purified by magnetic bead separation (EasySep CD4^+^ cell negative selection before [StemCell Tech, France]). To obtain naïve cells, CD4 T cells were stained with anti‐CD4^APCcy7^, anti‐CD25^PE^, anti‐CD44^PerCPcy5.5^, anti‐CD62L^BV421^, and sorted using FACSAria III. Cells were cultured in complete RPMI at a concentration of 1 × 10^6^ cells/mL in 96‐well plates coated with anti‐CD3ε at 5 μg/mL at 37°C and 5% CO^2^. All the cytokines and antibodies added were supplied by eBioscience (San Diego, CA, USA): Th0: anti‐CD28 (1 μg/mL), Th1: anti‐CD28 (1 μg/mL), anti‐IL‐4 (5 μg/mL), and rmIL‐12 (10 ng/mL); Th2: anti‐CD28 (1 μg/mL), anti‐IL‐12 (5 μg/mL), anti‐IFN‐γ (5 μg/mL), and rmIL‐4 (20 ng/mL). Cells were removed from activation after 3 days and expanded in rIL‐2 (20 ng/mL) for three more days in presence of specific recombinants.

### In vivo papain immunization and cell isolation from airway and lung

Mice were exposed to 25 μg/mL of papain protease (Sigma) in PBS or control 25 μL PBS in two doses (Supporting Information Fig. 2A). The solution was applied drop wise to the nose while mice were under isoflurane‐induced aaesthesia. BAL were collected by cannulating the trachea and lavaging the lungs four times with 1.0 mL of PBS + 0.01% EDTA. The BAL cells were pelleted, washed, and counted for further analysis. BAL supernatants were stored for cytokine analysis. Lung tissue was mechanically chopped and incubated al 37°C for 30 min in DMEM medium containing DNAse 0.5 mg/mL and Liberase 250 μg/mL (Roche, Basel, Switzerland). Cells were prepared for RNA extraction, flow cytometry analysis, or mechanically homogenized to obtain whole lung supernatants for cytokine analysis.

Lung lobes were isolated and fixed in phosphate‐buffered formalin (4% v/v) and sectioned for H&E and PAS staining performed by Histopathology, Great Ormond Street Hospital. Semiquantitative histological assessment was performed blind by two independent observers to score for cellular infiltration of the airways and mucous production. H&E staining was scored for cellular infiltration: 0, normal aspect; 1, mild infiltration around the airway; 2, moderate infiltration around the airway; 3, strong infiltration around the airway; and 4, severe infiltration around the airway and extravasation, majority of airway involved. PAS staining was scored for mucous production: 0–1, minimal; 1–2, moderate; and 2–3, severe. Pictures were photographed by Zeiss AxioCam digital camera with Zeiss Axioplan (NDU) Microscope, 20× Objective lens (Plan‐Neofluar/0.5NA) and 40× Objective lens (Plan‐Neofluar/0.75NA) and acquired by software AxioVision v4.8 (Zeiss) and analyzed using ImageJ software.

### Enzyme‐linked immunosorbent assay

IFN‐γ, IL‐4, IL‐13, and IL‐27 cytokines were measured by Ready‐Set‐Go! Kits (eBioscience), according to the manufacturer's instructions. IL‐6, IL‐17, IL‐5, IL‐10, and TNF‐α were measured by Firefly multiplex immunoassay mouse kit (Abcam, Cambridge, UK) following manufacturer's instructions.

### Quantitative reverse transcriptase polymerase chain reaction

RNA was extracted using Absolutely RNA miniprep kit (Agilent) or the PicoPure kit (Applied Biosystems, USA). cDNA was synthesized using High Capacity cDNA reverse transcription kit (Applied Biosystems). cDNA samples were analyzed on the iCycler (Bio‐Rad Laboratories, Hercules, CA, USA) using SYBR Green Supermix (Bio‐Rad) following manufacturer's guidelines. RNA levels obtained from each sample were measured relative to the housekeeping gene *Hprt*, as described 42. Primers were purchased from Quantitec (Qiagen, Venlo, Netherlands).

### Microarray and RNA‐sequencing data analysis

For microarray, RNA was extracted using the PicoPure RNA Isolation Kit (Applied Biosystems) according to the manufacturer's instructions from CD4^+^ T‐cells. Microarrays were performed by UCL Genomics on the Affymetrix Mouse Gene 1.0 ST Platform (GPL6246) using standard Ambion (Invitrogen, USA) chemistry as described 43. Data were analyzed as described 43, using univariate hypothesis testing by empirical‐Bayes moderated *t*‐statistics, followed by removal of false positives by the false discovery rate (FDR) method to identify DEG with fold change greater than two, which were then filtered for those with a minimum range of at least 250 units (RMA normalized nonlog value) to identify 920 DEG. CCA was carried out as previously described 39, 44, 45. We used the CCA function of the CRAN package library "vegan" for the calculations. All microarrays are publically available (GEO: GSE90494).

For RNA sequencing, naive CD4^+^ T cells from WT spleen were cultured in skewing conditions and RNA extracted as described above. RNAseq was carried out as described 46 in Th0‐, Th1‐, and Th2‐activated CD4^+^ T cells at 4‐h time points during 24 h after activation. RNA was sequenced by UCL Genomics on the Illumina Next Seq 500. Transcript expression was determined in reads Reads Per Kilobase of transcript per Million mapped reads (RPKM). The sequenced data are publically available (GEO: GSE93915).

### Statistics

Unpaired two‐tailed student's *t* test was used for statistical analysis and probabilities considered significant if *p* < 0.05(*), *p* < 0.01(**), and *p* < 0.001(***).

## Conflict of interest

The authors declare no financial or commercial conflict of interest.

**Abbreviations**
BAL: bronchoalveolar lavage
CCA: canonical correspondence analysis
DEG: differentially expressed gene
Hh: Hedgehog
IFITM: IFN‐inducible transmembrane
mLN: mediastinal lymph nodes
mDC: myeloid dendritic cell
PAS: periodic acid–Schiff

## Supporting information

Peer review correspondence

Supplementary Table 1: Genes of interest from intersection of DEG with 1000 genes that contributed most to PC1
Supplementary Figure 1. *Ifitm* family in peripheral T cells
Supplementary Figure 2. Cellular infiltration after papain sensitization
