# Supplementary material for: IFITM proteins drive type 2 T helper cell differentiation and exacerbate allergic airway inflammation
Source: Eur J Immunol. 2018 Nov 9;49(1):66–78. doi: 10.1002/eji.201847692 (PMC6396086; doi:10.1002/eji.201847692)
Supplement: Supplementary file 2 — Supplementary Table 1: Genes of interest from intersection of DEG with 1000 genes that contributed most to PC1 Supplementary Figure 1. Ifitm family in peripheral T cells Supplementary Figure 2. Cellular infiltration after papain sensitization [file EJI-49-66-s002.pdf]

European Journal of Immunology

Supporting Information
for
DOI 10.1002/eji.201847692

Diana C. Yánez, Hemant Sahni, Susan Ross, Anisha Solanki, Ching-In Lau, Eleftheria Papaioannou, Alessandro Barbarulo, Rebecca Powell, Ulrike C. Lange, David J. Adams, Martino Barenco, Masahiro Ono, Fulvio D'Acquisto, Anna L. Furmanski and Tessa Crompton

IFITM proteins drive type 2 T helper cell differentiation and exacerbate allergic airway inflammation

© 2018 The Authors. *European Journal of Immunology* published by WILEY-VCH Verlag GmbH & Co. KGaA, Weinheim.

Tmem71	Athl1	Morc3	Ndufc2	Arhgap4
Entpd4	**Cd27**	Cnot3	Snord68	Tmem209
Snca	Dennd2d	Airn	Atf7ip	Rps24
Pde6d	Apobec3	Ap3m1	Zfp831	Psmb3
Trim33	Zfp429	Samd9l	Calcrl	Clic1
Slc16a5	Ms4a6b	Cab39l	Rab37	Hdac1
Usp18	Klhl20	Rbl2	Stra13	**Stat1**
Fam78a	Tespa1	Nop10	Fkbp3	Il2ra
Tbxa2r	Uprt	Gm6907	Trim59	Trmt112
Pot1a	Ccdc53	Tspo	Cnot4	Ddx58
Slfn1	Nab2	Dpy30	Taf1d	Fam48a
Ska2	Snord47	Pigp	Mapk3	Cox7b
Nfe2l2	Dhrs11	Mcee	Add3	Dennd2d
Trav14-1	Zfml	Tgtp1	Ppp3cb	Glrx2
AI467606	Midn	Ndufb4	Arhgap9	Akt3
Ms4a4b	Fdft1	Herc6	Zfp141	Idnk
D14Ertd449e	Gpx4	Trim56	Cd3d	S100a11
Ttc13	Ifit3	Papola	Eif1b	Nlk
1110038D17Rik	Mudeng	Chmp5	S1pr4	Vps25
H2-T24	Casp6	Ndufs4	Larp4b	AY036118
Orc5	Tmc8	Gimap8	Vps35	Eif4ebp2
Klhdc5	Themis	Prkd2	Cd3g	Ehd3
Lfng	Lpar6	Apbb1ip	Exosc8	Ndufv2
Golph3l	Rps27a	Gm10136	Setx	Smek2
LOC100044193	Irgm2	Ift80	Mcmbp	Rps7
Ost4	Papolg	Psmb9	Psme1	Adnp
Zfp273	A2ld1	Psmd5	Phf12	Bcl11b
Clec2d	Lims1	Rps27l	Marf1	Rasgrp1
Rpp38	Casp8	Commd3	Uqcrh	Psmb3
Pcmtd2	Ube3a	Cpne1	Pank2	Spcs1
Tspan32	Tmem173	Twf2	Timm8b	Prl14l
Mina	Ifi47	Rnf38	Kbtbd11	Pydc3
Tbc1d10c	Zfp386	Gimap4	Ift20	Flt3l
Senp7	Cdkn2aipnl	Acss1	Ubl7	Mkl1
Zfp217	Rbl1	Psenen	Ikzf1	Trp53

Supplementary Table 1: Genes of interest from intersection of DEG with 1000 genes that contributed most to PC1

The intersection of the top 1000 genes contributing to differences on PC1. The list included 175 genes that are involved in various biological processes. Genes in bold are known to be part of the Th1 differentiation pathway.

Supplementary Figure 1

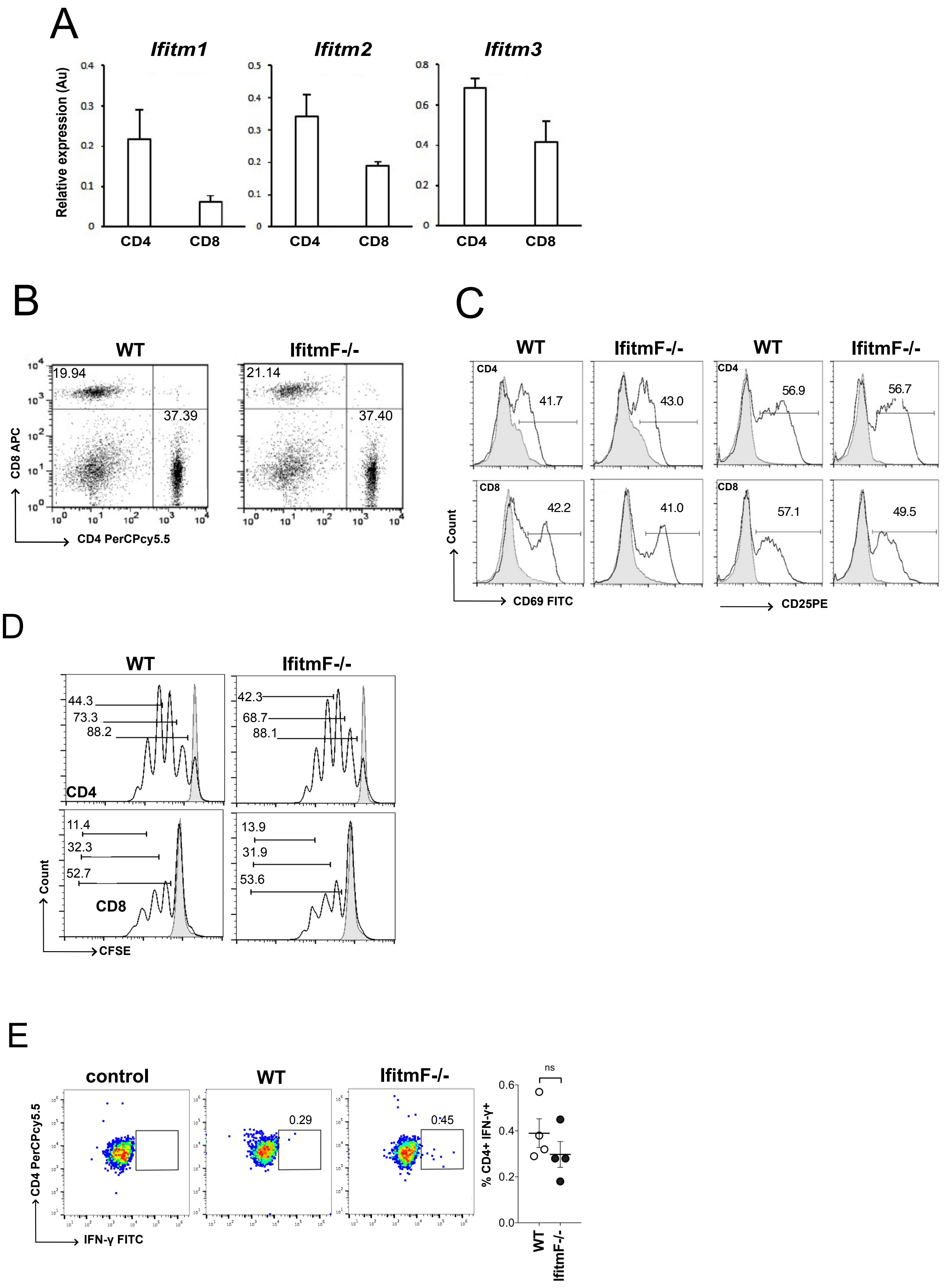

Supplementary Figure 1. ***Ifitm*** **family in peripheral T cells**

(**A**) (Q)RT-PCR showing mean expression of *Ifitm1-3* genes in three independent experiments of Facs sorted CD4 and CD8 T-cells from spleen, relative to *Hprt*. Units are arbitrary (Au). Error bars show SEM. (**B**) Dot plot: representative staining of CD4 and CD8 in *IfitmF*-/- (n=5) and WT (n=5) littermate lymph node, giving the percentage of each population. (**C**) Splenocytes from *IfitmF*-/- and WT mice were activated with anti-CD3 and anti-CD28 in vitro, and expression of CD69 and CD25 determined by flow cytometry. Histograms show expression of CD69 at 4h and CD25 at 40h after activation in a representative experiment. No significant differences between WT (n=5) and *IfitmF*-/- (n=5) in mean percentage of CD69 or CD25 expression on CD4 or CD8 T-cells. (**D**) Histograms show CSFE labelling in CD4 and CD8 splenocytes treated with anti-CD3/CD28 for 72h, in a representative experiment of 5 independent experiments. Markers indicate the proportion of cells that have completed one, two or three cell divisions. **(E)** Dot plots show CD4 against intracellular IFNγ staining on purified naïve CD4 cells from WT and *IfitmF*-/- that were cultured for 3 days in Th0 conditions. The percentage of cells in the region is given. Control plot shows the negative control for the cytokine staining (where the anti- IFNγ antibody was omitted from the staining). Graph shows mean ± SEM percentage of cells that stained positive for CD4 and IFN-γ.

Supplementary Figure 2

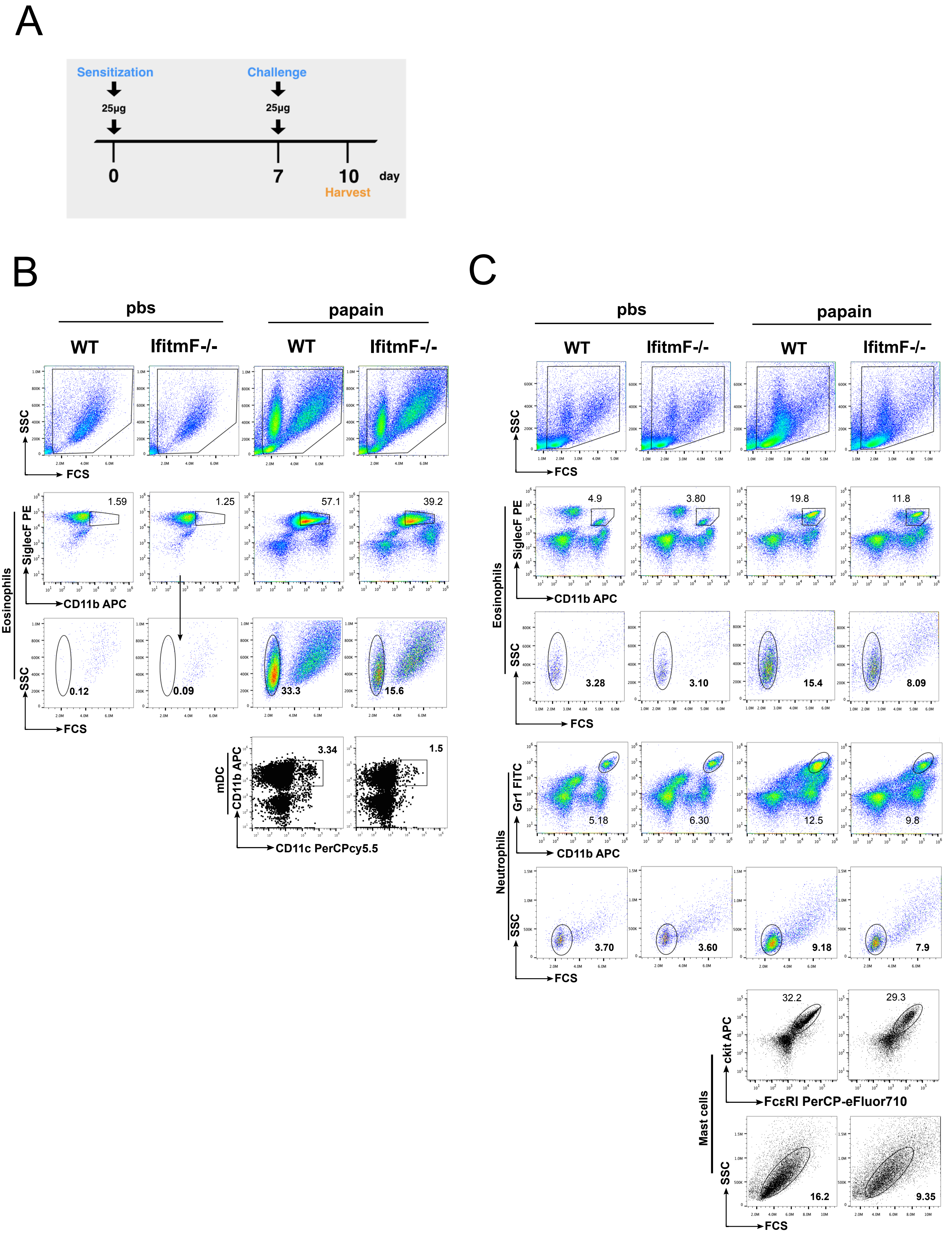

Supplementary Figure 2. Cellular infiltration after papain sensitization

(**A**) Graph shows the experimental protocol to induce allergic asthma. WT and *IfitmF-/-* mice were given intranasal application of either PBS or papain in PBS (25µg/ml) on indicated days. These mice were then analyzed 3 days after the final intranasal application. (**B**-**C**) Flow cytometry strategy used to analyse cellular infiltration. (**B**) Plots show FSC and SSC, and expression of leukocyte markers to gate on leucocyte populations of eosinophils ($CD11b^{+}SiglecF^{+}FSC^{low}SSC^{int}$) and mDC ($Gr1^{-}CD317^{-}$ $CD11b^{+}CD11c^{+}$) in BAL. Numbers on flow cytometry plots refer to percentage of populations, and bold numbers indicate the percentage of live gate. (**C**) Plots show FSC and SSC, and expression of leukocyte markers to gate on leucocyte populations of eosinophils ($CD11b^{+}SiglecF^{+}FSC^{low}SSC^{int}$); ($CD11b^{hi}CD11c^{-}Ly6g^{+}$ $FSC^{low}SSC^{low}$) and mast cells ($ckit^{+}Fc\varepsilon RI^{+}FSC^{int}SSC^{int}$) in lungs. Numbers on flow cytometry plots refer to percentage of populations, and bold numbers indicate the percentage of live gate
