# Supplementary material for: IFITM proteins drive type 2 T helper cell differentiation and exacerbate allergic airway inflammation
Source: Eur J Immunol. 2018 Nov 9;49(1):66–78. doi: 10.1002/eji.201847692 (PMC6396086; doi:10.1002/eji.201847692)
Supplement: Supplementary file 1 — Peer review correspondence [file EJI-49-66-s001.pdf]

European Journal of Immunology

IFITM proteins drive type 2 T helper cell differentiation and exacerbate allergic airway inflammation

Diana Yanez, Hemant Sahni, Susan Ross, Anisha Solanki, Ching-In Lau, Eleftheria Papaioannou, Alessandro Barbarulo, Rebecca Powell, Ulrike Lange, David Adams, Martino Barenco, Masahiro Ono, Fulvio D'Acquisto, Anna Furmanski, Tessa Crompton

Correspondence: Dr. Crompton, Tessa, 30 Guilford Street, London, WC1N 1EH, United Kingdom

---

Review Timeline:	Submission date:	10-May-2018
	First Editorial decision:	4-Jul-2018
	Revisions received:	13-Sep-2018
	Accepted:	17-Oct-2018

Handling Executive Committee member: Prof. Francesco Annunziato

Please note that the correspondence below does not include the standard editorial instructions regarding preparation and submission of revised manuscripts, only the scientific revisions requested and addressed.

First Editorial Decision
04-Jul-2018

Dear Dr. Crompton,

Manuscript ID eji.201847692 entitled "The IFITM protein family provide negative feedback on IFN-Î³ signalling in CD4 T-cells" which you submitted to the European Journal of Immunology has been reviewed. The comments of the referees are included at the bottom of this letter. Please accept again our sincere apologies for a delay in the peer review. As mentioned, one of the referees who initially agreed never submitted the report, and we had to seek a help of another referee.

You will note that the referees have noted that the number of experiments as well as the number of samples/replicates/mice per experiment is not clearly stated. Because this information is lacking, it is difficult to assess the reproducibility of experiments performed and you are strongly encouraged to repeat experiments if performed only once to ensure that data shown are reproducible and statistically robust. A revised version of your manuscript that takes into account the comments of the referees will be reconsidered for publication. Should you disagree with any of the referees' concerns, you should address this in your point-by-point response and provide solid scientific reasons for why you will not make the requested changes.

You should also pay close attention to the editorial comments included below. **In particular, please edit your figure legends to follow Journal standards as outlined in the editorial comments. Please also show full gating strategy, axis fluorochrome labels and scaling in all flow cytometry plots. Failure to do this will result in delays in the re-review process.**

Please note that submitting a revision of your manuscript does not guarantee eventual acceptance, and that your revision will be re-reviewed by the referees before a decision is rendered.

If the revision of the paper is expected to take more than three months, please inform the editorial office. Revisions taking longer than six months may be assessed by new referees to ensure the relevance and timeliness of the data.

Once again, thank you for submitting your manuscript to European Journal of Immunology and we look forward to receiving your revision.

Yours sincerely,
Nadja Bakocevic

On behalf of
Prof. Francesco Annunziato

Dr. Nadja Bakocevic
Editorial Office
European Journal of Immunology
e-mail: ejied@wiley.com
www.eji-journal.eu

****

Reviewer: 1

Comments to the Author
In the manuscript by Yanez et al the authors claim that interferon-inducible transmembrane (IFITM) family proteins are inhibiting IFNg signalling and Th1 differentiation and favor Th2 differentiation. This is somewhat contradictory to the finding that one member, IFTIM3 (as cited by the authors) is required for antiviral protection and CD8 T cell responses which is rather dependent on Th1 responses. They analyze

European Journal of Immunology

the effect of the gene ko ex vivo, in in vitro differentiation cultures under neural and polarizing conditions and in the papain induced Th2 allergy/asthma in vivo model.

In general the work is well done and seem to support the idea that Th1 development is favoured in the absence of IFITM. However, some specific points to address remain.

Specific points:

1. Figure 1: Although the data suggest that there is a bias towards increased Th1 responses and decreased Th2 responses in IFITM family ko mice the effects are not very strong and not unambiguous. Especially it is remarkable that despite a high number of differentially regulated genes between ko and WT T cells the relevant Th1 and Th2 hallmark key transcription factors Tbet and GATA3 or key effector cytokines like IFNg or signaling components IL-12Rbeta chain/STAT4 and IL-4 are not only minimally affected. Most of the differences are only marginal. To confirm that IFITM affect Th1 versus Th2 differentiation the authors should focus on the lineage defining transcription factors and effector molecules (Tbet, GATA3, IL-12Rbeta chain, IL-4, IFN-g) whereas CD27, STAT1 or CD154 are not really hallmark genes of Th1 differentiation. The differential expression should also be confirmed by RT PCR and on the protein level since the differences are mostly marginal.

2. The authors do not demonstrate that IFITM really affects IFN-g signaling as claimed in the title. What about the phosphorylation of STAT1 or STAT4 which required for Th1 differentiation. No functional data are provided that IFN signaling is affected. Also it is known that IL-12Rbeta chain is upregulated upon IFNg signaling during Th1 differentiation. Is this gene not differentially expressed?

3. Figure 2 Here the authors analyse the expression of the master transcription factors following in vitro stimulation (this is missing ex vivo see point 1). The results show increased Tbet expression in IFITM ko under neutral and Th1 conditions.

In general it would be nice to see the gating strategy and dot plots (e.g. Tbet versus GATA3) for the various markers (suppl figure). They also claim differential expression of CXCR3 but the histograms do not show that. There are not 2 populations, rather the whole population is shifted, this is better characterized by the mfi of the population than by the % of positive cells.

For figures 2 B-E the authors show fold increase versus WT. How is that calculated (normalized)? Would it not be more appropriate to show the frequencies or mfi etc. of the WT versus ko (like in 2 G)? Actually the differences for the bar diagrams look much weaker as compared to the shown dotplots. Are the dot plots really representative? This analysis has to be redone and made more clear.

4. Figure 3: The frequencies of IFNg+ T cells and IFNg production are anaylzed. The data for the Th0

culture are missing. Also here the observed differences are rather low for the intracellular cytokine staining.
In particular the data from the Th2 cultures look not convincing (very weak/absent cytokine staining). Either the culture conditions or the IL-4/13 staining have to be improved. Also Tbet/GATA3 can be counterstained with cytokines in vitro cultured cells to confirm the point. Figure 2 and 3 should be combined using all analysis tools for all culture conditions.

5. It remains unclear at how the IFTIM proteins mediate the effect (signaling, transcription,...). The mechanistic details should be addressed in particular regarding the reciprocal regulation of the two main family members of interest (IFITM 2 upregulated and 3 down regulated) following T cell activation. Also the claim that IFITM proteins influence IFN-g signalling activation has not been demonstrated.

6. Figure 4&5: I am not an expert for this in vivo Th2 model. However the data seem tosupport the claim that Th2 responses are decreased in ko animals. The differences however seem to be rather small sometimes, is that typical for this model? Can both be combined in one figure?

7. Figure 6. The authors analyzed the ko of IFITM3 only, which has been shown to susceptible to viral infections. This had no effect on the Th1/2 bias analyszed. To me the conclusions are not really clear. Does IFITM 2 mediate all effects alone (ko available?) are both redundant or is it another IFITM. Is IFITM mediating antiviral protection independent of the action on Th1/2?

Reviewer: 2

Comments to the Author
Yanez et al use murine CD4 T cells devoid of Ifitm family members and show that these cells preferentially differentiate towards a Th1 phenotype. Using a murine model of allergic asthma the authors show that knockout of Ifitm family members reduces the severity of allergic airways disease. Overall, the results are novel and the paper is well written. There are some issues with data representation.

While deletion of all Ifitm family members reduces the induction of allergic airway disease, in the last chapter of the manuscript the authors present data showing that knockout of Ifitm3 alone does not affect Th polarization nor the induction of allergic airway disease. In the discussion, the authors mention that most likely Ifitm2 is the key family member that is required for Th2 differentiation and inhibition of Th1 differentiation. Are Ifitm2 KO mice available and is it possible to provide any data for this?

European Journal of
Immunology

Issues with data representation:

An interesting finding is that deletion of Ifitm family members reduces the induction of allergic airway disease. In Figure 4I three histological sections are shown without any label. Are these WT and Ifitm KO? Why three? On the right side, there is a barplot showing a score. What is this score and what does it mean. I could not find a description in the text or in the figure legends. Is this a strong effect? Does it have implications for the treatment of asthma patients? Same for Figure 4J.

Figure 1A: RNA Seq data from Th0, Th1, and Th2 cells are shown. The y-axis is labeled with Transcript per million kilobases. How does this relate to an RPKM value that is described in the M&Ms and is typically used to express RNA Seq data? In my opinion, colors would help the reader to distinguish the different lines in the graph. The figure legend does not indicate the number of replicates.

Figure 1B shows the Log2 fold change but it is labeled with fold change. It is not clear to me what the authors mean by â€œmean intensityâ€□. Is it the mean of all samples?

Figure 1C: It would be helpful to indicate sample numbers (n=?).

Figure 1F: The legend says histogram but the authors show a barplot. Some differences are marked with a star but I could not find a description of the statistical test that was used nor the sample number (n=?).

Figure 2: In Figure 1B the barplot is labeled with â€œRelative to WTâ€□ and WT is set to 1. However, in Figure 1D the barplot is labeled in the same way but most likely the absolute percentage is shown (different scale).

Figure 3B,D and F: The barplot shows WT and IfitmF. I assume the authors meant IfitmF -/-?

Figure 4B: “Mean number of BAL”. BAL is the lavage, so it is not clear to what the number refers to.

<u>First Revision – authors’ response</u>

Reviewer: 1

Comments to the Author

In the manuscript by Yanez et al the authors claim that interferon-inducible transmembrane (IFITM)

European Journal of Immunology

family proteins are inhibiting IFNg signalling and Th1 differentiation and favour Th2 differentiation. This is somewhat contradictory to the finding that one member, IFTIM3 (as cited by the authors) is required for antiviral protection and CD8 T cell responses which is rather dependent on Th1 responses.

Response: The findings presented in our manuscript are indeed entirely different from previous studies and therefore highly novel, but they are not contradictory to the published reports that IFITM3 is required for memory CD8+ T cell response on influenza infection (Ref. 14, Wakim et al 2013; Ref. 15 Infusini et al 2015). The previous published reports show that when mice are infected with influenza, IFITM3 enhances the survival of lung-resident CD8 T cells and the mechanism for its action is by protecting dendritic cells from infection by the influenza virus and thereby enabling them to effectively prime CD8 T cells. Thus, the two cited publications show the consequences of IFITM3's role in cellular resistance to viral infection, but do not demonstrate a direct T cell-autonomous function for IFITM3 in T cell differentiation or show that IFITM3 promotes a Th1 response. We have in fact stated in the introduction that Ifitm3-/- are highly susceptible to influenza infection and cited those publications, as well as others, but stated that their role in adaptive immunity per se has not been explored (Page 3, last paragraph).

They analyze the effect of the gene ko ex vivo, in in vitro differentiation cultures under neural and polarizing conditions and in the papain induced Th2 allergy/asthma in vivo model. In general the work is well done and seem to support the idea that Th1 development is favoured in the absence of IFITM. However, some specific points to address remain.

Specific points:

1. Figure 1: Although the data suggest that there is a bias towards increased Th1 responses and decreased Th2 responses in IFITM family ko mice the effects are not very strong and not unambiguous. Especially it is remarkable that despite a high number of differentially regulated genes between ko and WT T cells the relevant Th1 and Th2 hallmark key transcription factors Tbet and GATA3 or key effector cytokines like IFNg or signaling components IL-12Rbeta chain/STAT4 and IL-4 are not only minimally affected. Most of the differences are only marginal. To confirm that IFITM affect Th1 versus Th2 differentiation the authors should focus on the lineage defining transcription factors and effector

European Journal of Immunology

molecules (Tbet, GATA3, IL-12Rbeta chain, IL-4, IFN-g) whereas CD27, STAT1 or CD154 are not really hallmark genes of Th1 differentiation. The differential expression should also be confirmed by RT PCR and on the protein level since the differences are mostly marginal.

Response: The conclusion that the IFITM-deficient resting CD4 cells from the spleen had a Th1-like pattern of expression came from canonical correspondence analysis (CCA), looking at the overall pattern of gene expression in the entire genome, not from the expression values for individual genes. We used CCA to compare our genome-wide transcriptome data to datasets from murine T-cells that have been differentiated under Th1 and Th2 skewing conditions for 6 days (Fig. 1G). CCA is a multivariate analysis that allows the comparison of experimental transcriptome data with datasets from other laboratories (Ono et al., 2014, Furmanski et al 2013, Solanki et al 2018). CCA thus looks at the overall pattern of gene expression in a quantitative manner and provides a score of how 'Th1-like' or 'Th2-like' the overall pattern of transcription in the cells is. This analysis clearly showed that overall the IFITM-deficient CD4 cells had a pattern of transcription that was more biased towards that of Th1-cells than the WT CD4 cells. In our microarray analysis, we do not wish to suggest that Cd27, Stat1 and Cd154 are the hallmark genes of a Th1 response, but rather the fact that the intersection between the genes identified by principal component analysis and the differentially expressed genes identified Cd27 and Stat1 and the fact that they are involved in Th1 responses then led us to look in more detail at other genes associated with Th1 and Th2 responses (Figure 1F). We have changed the text to explain this more clearly (Page 5, second paragraph, highlighted). The fact that many other Th2-associated and Th1-associated genes were also differentially expressed then led us to carry out the CCA to assess the overall pattern of expression of Th1 and Th2 genes. We have now changed the text to make this point clearer (Page 5, bottom of page, highlighted).

In the results section we point out that the expression of Tbx21 (which encodes Tbet) is not different in the resting CD4 cells from WT and IFITM-deficient mice (page 5, 2nd paragraph and Figure 1F). We believe this is important, as we only detect changes in Tbet/Tbx21 expression when T cells were activated and this is shown at both protein level by flow cytometry and gene level by QRT-PCR (Figure 2B, C and E; Figure 3B). Thus, we do not claim that resting CD4 cells from the spleen are already differentiated into Th1 cells, but rather that they have a bias towards Th1-differentiation when induced to differentiate.

2. The authors do not demonstrate that IFITM really affects IFN-g signaling as claimed in the title. What

about the phosphorylation of STAT1 or STAT4 which required for Th1 differentiation. No functional data are provided that IFN signaling is affected. Also it is known that IL-12Rbeta chain is upregulated upon IFNg signaling during Th1 differentiation. Is this gene not differentially expressed?

Response: We accept the criticism that our title is not appropriate because we do not really show a direct effect on IFNg signalling per se. We have therefore changed the title to 'The IFITM protein family influence CD4 T helper cell differentiation and Th1/Th2 polarization'. The gene encoding the IL-12beta chain was not upregulated in our microarray dataset from resting IFITM-deficient CD4 cells.

3. Figure 2 Here the authors analyse the expression of the master transcription factors following in vitro stimulation (this is missing ex vivo see point 1).

Response: We did not see a reduction in Tbx21 expression ex vivo (Fig 1F) and protein levels of Tbet and Gata3 are very low/below detection in naïve CD4 cells, so we have not shown them. We have now included both Tbet and Gata3 protein levels by flow cytometry after activation in vitro in Th0, Th1 and Th2 skewing conditions at 72 hours (Figure 2C and D) and we have shown Tbet-against-IFNg staining and Gata3-against-IL4 staining in the cultures at 6 days (Fig. 3B and C).

The results show increased Tbet expression in IFITM ko under neutral and Th1 conditions. In general it would be nice to see the gating strategy and dot plots (e.g. Tbet versus GATA3) for the various markers (suppl figure).

Response: We have included the gating strategy in Fig. 2B.

They also claim differential expression of CXCR3 but the histograms do not show that. There are not 2 populations, rather the whole population is shifted, this is better characterized by the mfi of the population than by the % of positive cells.

Response: As suggested by the Reviewer we have now also shown the increase in MFI of anti-Cxcr3 staining (Figure 2F). We agree with the Reviewer that the histogram shows a shift, with a shoulder rather than a clearly separated distinct peak. Therefore, we have also shown the percentage of cells that fall within the marker shown in the histogram, and this is now explained in the figure legend.

For figures 2 B-E the authors show fold increase versus WT. How is that calculated (normalized)? Would it not be more appropriate to show the frequencies or mfi etc. of the WT versus ko (like in 2 G)? Actually the differences for the bar diagrams look much weaker as compared to the shown dotplots. Are the dot plots really representative? This analysis has to be redone and made more clear.

Response: We have improved our presentation of these experiments, so that rather than showing the fold increase, we now show the actual percentage of cells that stain positive under Th0, Th1 and Th2 conditions for WT and KO (Fig 2B to D).

4. Figure 3: The frequencies of IFNg+ T cells and IFNg production are anaylzed. The data for the Th0 culture are missing.

Response: We have added the facs plots for IFNg staining in Th0 cultures to supplementary Figure 1E. Levels of IFNg were very low under Th0 conditions and we did not observe statistically significant differences.

Also here the observed differences are rather low for the intracellular cytokine staining. In particular the data from the Th2 cultures look not convincing (very weak/absent cytokine staining). Either the culture conditions or the IL-4/13 staining have to be improved. Also Tbet/GATA3 can be counterstained with cytokines in vitro cultured cells to confirm the point.

Response: We have added the facs plots for ic cytokines and transcription factors under Th1 and Th2 conditions to confirm the results (Fig. 3B-C). In both our published work in murine experiments (eg Furmanski et al JI 2013) and in this study, we have consistently observed lower anti-IL4 staining than anti-IL13 or anti-IFNgamma staining, but our results are consistent and statistically significant.

Figure 2 and 3 should be combined using all analysis tools for all culture conditions.

Response: We were unable to combine Figure 2 and 3 because of the volume of experimental data, as the figure became too busy to put on one page. However, we have included both protein expression (Flow cytometry), Gene expression (QRT-PCR) and ELISA data for cytokine levels and these data are all

consistent.

5. It remains unclear at how the IFTIM proteins mediate the effect (signaling, transcription,...). The mechanistic details should be addressed in particular regarding the reciprocal regulation of the two main family members of interest (IFITM 2 upregulated and 3 down regulated) following T cell activation.

Response: We agree with the Reviewer that it would be very interesting to know mechanistically how the IFITM proteins are having their effect on Th differentiation/polarization and also to know why IFTM2 is upregulated and IFITM3 is downregulated. As this is the first evidence that IFITM proteins directly influence T cell function our study is highly novel and important, and we believe that it is beyond the scope of this manuscript to also address the mechanism. Indeed, in the case of the IFITM family's function in providing cellular resistance to viral infection, the mechanism is still unknown and different mechanisms have been proposed for different family members and for different viruses (see cited references 7 to 11). In the discussion of our manuscript we have included a paragraph that discusses the mechanism in the context of the influence of IFITM proteins on membrane fluidity.

Also the claim that IFITM proteins influence IFN-g signalling activation has not been demonstrated.

Response: We accept this criticism and we have therefore changed the title to 'The IFITM protein family influence CD4 T helper cell differentiation and Th1/Th2 polarization'.

6. Figure 4&5: I am not an expert for this in vivo Th2 model. However the data seem to support the claim that Th2 responses are decreased in ko animals. The differences however seem to be rather small sometimes, is that typical for this model? Can both be combined in one figure?

Response: The papain protocol that we used as a model of allergic airway disease driven by Th2 cells is widely accepted and used (see page 8 and cited references 26 to 29) and the effects we observe are within expected ranges for this model. We were unable to put all the data from the papain model in the IFITMfamily-deficient animals into one figure because the figure became too busy but we have added additional figures in Sup. Fig 2, in which we show the cartoon describing the protocol and the cellular infiltration in BAL and Lung in PBS and papain conditions from WT and IfitmF-/-.

7. Figure 6. The authors analyzed the ko of IFITM3 only, which has been shown to susceptible to viral infections. This had no effect on the Th1/2 bias analyszed. To me the conclusions are not really clear. Does IFITM 2 mediate all effects alone (ko available?) are both redundant or is it another IFITM. Is IFITM mediating antiviral protection independent of the action on Th1/2?

Response: The data shown in Figure 6 indeed show that we do not detect an influence on Th1/Th2 differentiation or the T-cell response in allergic asthma when IFITM3 alone is deleted. As we have discussed in the Discussion, these experiments indicate that IFITM2 is the most likely candidate for the observed effects on Th1/Th2 differentiation in the entire family KO. However, we include the caveat that we cannot exclude a synergistic or additive effect between the family members. We make no connection between the role of IFITMs in antiviral protection, in which all family members have been described to have different roles and protect against different viruses by different suggested mechanisms, and their influence on Th differentiation, but as mentioned in the discussion an influence on membrane fluidity might affect both processes.

Reviewer: 2

Comments to the Author
Yanez et al use murine CD4 T cells devoid of Ifitm family members and show that these cells preferentially differentiate towards a Th1 phenotype. Using a murine model of allergic asthma the authors show that knockout of Ifitm family members reduces the severity of allergic airways disease. Overall, the results are novel and the paper is well written. There are some issues with data representation.

While deletion of all Ifitm family members reduces the induction of allergic airway disease, in the last chapter of the manuscript the authors present data showing that knockout of Ifitm3 alone does not affect Th polarization nor the induction of allergic airway disease. In the discussion, the authors mention that most likely Ifitm2 is the key family member that is required for Th2 differentiation and inhibition of Th1 differentiation. Are Ifitm2 KO mice available and is it possible to provide any data for this?

Response: Unfortunately Ifitm2 KO mice are not available and although we accept that in the long term it

European Journal of Immunology

would be very interesting to assess these, we believe it is beyond the scope of our present study.

Issues with data representation:

An interesting finding is that deletion of Ifitm family members reduces the induction of allergic airway disease. In Figure 4I three histological sections are shown without any label. Are these WT and Ifitm KO?

Response: We have added the correct labels to the histological sections which are now shown in figure 4C and D.

Why three? On the right side, there is a barplot showing a score. What is this score and what does it mean. I could not find a description in the text or in the figure legends.

Response: We have added a full description of the scoring in the Materials and Methods and figure legends and we have labelled the bar charts to explain the score. We have scored for cellular infiltration and for mucous production and this is now explained fully.

Is this a strong effect? Does it have implications for the treatment of asthma patients? Same for Figure 4J.

Response: The histological differences are strong, and so in the long term it may have implications for human asthma patients, but we are hesitant to speculate on this at this early stage.

Figure 1A: RNA Seq data from Th0, Th1, and Th2 cells are shown. The y-axis is labelled with Transcript per million kilobases. How does this relate to an RPKM value that is described in the M&Ms and is typically used to express RNA Seq data?

Response: We apologise for this and agree that our labelling is confusing. We have changed the y-axis label in Fig1A and Fig2A to RPKM (Reads Per Kilobase of transcript per Million mapped reads). We have also defined this correctly in the M&M (highlighted on Page 17).

In my opinion, colours would help the reader to distinguish the different lines in the graph. The figure

legend does not indicate the number of replicates.

Response: We have added colours to Fig1A. For the time series experiments shown in Figure 1A, replicates were not carried out as the different time points serve as replicates to confirm expression patterns and trends (see Sahni et al 2015).

Figure 1B shows the Log2 fold change but it is labeled with fold change. It is not clear to me what the authors mean by “mean intensity”. Is it the mean of all samples?

Response: We have changed the Figure legends to explain the axes better. Both axes are on a Log2 scale. The mean intensity is the RMA normalized value, plotted on a Log2 scale. The fold change is the relative change in expression. This is now stated in the figure legend.

Figure 1C: It would be helpful to indicate sample numbers (n=?).

Response: We have added number of replicates to the figure legends shown on page 24.

Figure 1F: The legend says histogram but the authors show a barplot. Some differences are marked with a star but I could not find a description of the statistical test that was used nor the sample number (n=?).

Response: We have corrected this to state that it is a barplot and added sample number and statistical test to the Figure legend.

Figure 2: In Figure 1B the barplot is labeled with “Relative to WT” and WT is set to 1. However, in Figure 1D the barplot is labeled in the same way but most likely the absolute percentage is shown (different scale).

Response: We have changed Figure 2 to show the percentage of positive cells, as this was requested by the other Reviewer.

Figure 3B,D and F: The barplot shows WT and IfitmF. I assume the authors meant IfitmF -/-?.

Response: The reviewer is correct, and we have added the correct labels to the Figure.

Figure 4B: “Mean number of BAL”. BAL is the lavage, so it is not clear to what the number refers to.

Response: We have explained this in the Figure legend, as we are referring to the mean number of cells in the BAL.

Second Editorial Decision

17-Oct-2018

Dear Dr. Crompton,

It is a pleasure to provisionally accept your manuscript entitled "The IFITM protein family influence CD4 T helper cell differentiation and Th1/Th2 polarization" for publication in the European Journal of Immunology. For final acceptance, please follow the instructions below and return the requested items as soon as possible as we cannot process your manuscript further until all items listed below are dealt with.

Please note that EJI articles are now published online a few days after final acceptance (see Accepted Articles: https://onlinelibrary.wiley.com/toc/15214141/0/ja). The files used for the Accepted Articles are the final files and information supplied by you in Manuscript Central. You should therefore check that all the information (including author names) is correct as changes will NOT be permitted until the proofs stage.

We look forward to hearing from you and thank you for submitting your manuscript to the European Journal of Immunology.

Yours sincerely,
Ivan Shevchenko

on behalf of
Prof. Francesco Annunziato

Dr. Ivan Shevchenko
Editorial Office
European Journal of Immunology
e-mail: ejied@wiley.com

European Journal of Immunology

www.eji-journal.eu
